# Experiences of empathy and literary empathy between children using the LuDiCa (dialogic reading for deep comprehension)

**DOI:** 10.3389/fpsyg.2025.1725818

**Published:** 2025-12-10

**Authors:** Bianca da Nóbrega Rogoski, Eileen Pfeiffer-Flores

**Affiliations:** 1 Education Department of the Federal District, Brasília, Brazil; 2 Department of Psychology, University of Brasília, Brasília, Brazil

**Keywords:** empathy, dialogic reading, literary empathy, perspective taking, immigrant children

## Abstract

This study investigated the effects of the shared reading technique LuDiCa (Dialogic Reading for Deep Comprehension) on interpersonal and literary empathy among school-age children. In the LuDiCa method, a pre-reading analysis of the story leads to the preparation of open-ended questions that are used during shared reading with children. Unlike traditional dialogic reading, these questions are carefully designed to foster deep comprehension by guiding discussion around narrative functions. Narrative functions are what the story shows the reader through events, dialogues, descriptions and other elements, often implicitly. Twelve children participated in LuDiCa sessions of stories about immigrants and refugee children. LuDiCa significantly promoted expressions of both interpersonal empathy among participants and literary empathy toward immigrant and refugee characters. The findings suggest that interactive group reading sessions using the LuDiCa method can effectively enhance empathy, contributing to a deeper understanding of diverse human experiences.

## Introduction

Literary empathy involves understanding and vicariously experiencing the emotions and thoughts of characters in a narrative (Keen, 2007). In Psychology, it has been studied primarily for its impact on attitudes and behaviors toward others after reading, including perspective taking and empathy (e.g., Igartua et al., 2018; Johnson, 2012; Koopman, 2015; Vezzali et al., 2014). One reason for hypothesizing that literature can foster empathy is that narratives offer a safe way to engage with different realities and experiences, potentially helping readers broaden their understanding of human complexities.

Empathy has been identified as a concept lacking consensus in the literature (e.g., Cuff et al., 2014; Stein, 1989; Wiseman, 1996). One theoretical approach has been commonly used to study empathy, the Theories of Mind. They understand empathy from a dualistic perspective, assuming that one can only directly observe another person's behavior, not their inner experiences (Leudar and Costall, 2009). This framework has influenced contemporary psychological models of empathy, which typically divide it into cognitive empathy, the capacity to understand another's feelings; and affective empathy, the emotional state that arises in response to them (e.g., Bloom, 2016; Cuff et al., 2014).

In contrast, the perspective of this work is based on a relational perspective of empathy, in which it is considered as an interactive experience. Both the empathizer and the empathizee participate in this shared process, each perceiving and responding to the other's expressions and emotions (Pfeiffer-Flores and Rogoski, 2023). Thus, empathy is not confined to the mind of one individual but is manifested and understood through lived, reciprocal interaction (Pfeiffer-Flores and Rogoski, 2023).

Similarly, literary empathy appears in the interaction between the reader and the text. This is a borderline case of the concept of empathy, since there is no interaction with a real person, but rather with a character (Rogoski, 2024). However, this contact brings the possibility of safe emotional involvement, allowing for the expansion of personal experiences (Rogoski, 2024).

This expansion of individual perspectives has been highlighted as essential, particularly in the face of contemporary life characteristics such as individualism and tendencies toward superficial relationships (Bauman, 2004; Rifkin, 2009). This becomes even more evident in contexts involving intolerance, disrespect, and violence (e.g., contexts of sexism, racism, homophobia, ableism, etc.). Empathy, as the capacity to understand, empathize, and be available to the other based on their needs, is seen as essential in addressing these issues (e.g., Font et al., 2016; Pedersen, 2009; Tavares, 2022).

The National Literacy Trust, a UK institution that enhances reading development by supporting teachers, families and communities, conducts an annual survey about reading, writing and listening habits of children and adolescents from 5 to 18 years old. The 2024 survey included questions about connections between empathy and reading and the results revealed that one-third of young readers reported that reading helps them understand different perspectives and cultures, indicating the role of literature in promoting social empathy among youth. Also, 20.7% reported they read to feel more connected with the world (Clark et al., 2024).

Empathy is sometimes analyzed in terms of the influences of the context and social cultural factors. Zurek and Scheithauer (2017) and Hoffman (2000) states that people who have been to similar situations can feel empathy to one another more easily. In this sense, empirical studies have also found results suggesting that reading narratives can foster empathy. Vezzali et al. (2014), for example, investigated the effect of reading the *Harry Potter* novels on the stigmatization of minority social groups. In this sense, Italian children participated in reading sessions of passages from Harry Potter, either related to prejudice (experimental group) or not related (control group). The 58 children responded to a scale about personal attitudes toward immigrants and completed questionnaires regarding their identification with the characters Harry Potter (the protagonist, hero of the story) and Voldemort (the villain of the story). Stigmatization was lower in the experimental group, but only for children who identified with Harry Potter and did not identify with Voldemort.

Igartua et al. (2018), in turn, investigated the effect of identification with characters on the attitudes of Spanish university students toward immigration. To do this, they used questionnaires that assessed attitudes toward immigrants, narrative transportation (immersion in the story), identification with the main characters, contact with Moroccan immigrants, and the degree of perceived realism of the stories read. Participants identified more with characters more similar to them, with an indirect effect on favorable attitudes toward immigration.

More recently, Shen et al. (2023) used the State Empathy Scale to compare the effects of similarity of style vs. identification of characters on empathetic responses. Participants wrote their personal stories and then read someone else's story with either a similar writing style or similar characters. Results showed that readers empathized better with characters they identified with, independently of the writing style.

A critic to this cultural perspective of empathy (e.g., Bloom, 2016) would question if this is really empathy, as it is not really a perspective taking if the empathizer is taking the perspective of someone who is experiencing a situation very similar to the one she/he is experiencing (or have experienced) her/himself. Also, as stated by Stein (1989) one can empathize with the sadness of a friend, even if she/he is very happy for a great news just received. In this work, as explained above, the theoretical perspective is that empathy occurs through interaction. Therefore, understanding the experiences and emotional state of the empathizee is possible even if the empathizer has not experienced something similar.

Another limitation of most of these studies is that empathy has been measured through self-reports (e.g., Igartua et al., 2018; Shen et al., 2023; Vezzali et al., 2014). Two studies (Johnson, 2012; Koopman, 2015) employed behavioral measures. In Johnson (2012), experimenters dropped pens to gauge participants' willingness to help after reading stories. Results showed that participants with higher scores of empathy and narrative transportation were significantly more likely to show prosocial behavior. In Koopman (2015), participants read expository vs. narrative texts about grieving and depression. Then, participants were given money for their participation and asked if they would donate it to charities aligned with the narrative themes. Thirty one of the 210 participants donated. Despite the small number, there was a significant difference between text genres, with participants who read narratives donating more.

While these studies used direct measures of empathy, they overlooked interactions during the shared reading of literary works. This omission is critical, as the impact of literary reading on empathy might be most evident during the actual shared reading experience. Recent research suggests that group settings may better explore this phenomenon by examining both reader-text and inter-participant interaction (e.g., Fernandez-Quintanilla, 2020; Kurcikova, 2019).

An illustrative example of the importance of examining real-time interactions during shared reading comes from Gosen et al. (2015) who used discourse analysis to assess interaction of 4 to 6-year-old children and their teachers during interactive reading sessions, focusing on moments when children engaged in problem-solving, specifically, when they attempted to find solutions to the characters' problems. Their results showed that teachers often needed only to describe the characters' situations for children to engage in problem-solving dialogues. This finding suggests that real-time interactions during shared reading can foster empathy, as children naturally engage in perspective-taking and seek solutions to characters' problems without direct prompting.

Still on the subject of real-time vs. offline assessments, Fernandez-Quintanilla (2020) used focus groups to assess adult readers' emotional reactions to two biographical narratives by Uruguayan author Eduardo Galeano after individual reading. However, she identified a significant drawback of her method: the discussion about the story took place only after reading. This gap she hypothesized, may have lead participants to forget specific passages or to rationalize their emotions. In contrast, discussing the text during reading, particularly in group settings, could elicit more spontaneous responses and reduce forgetfulness. The Dialogic Reading for Deep Comprehension (LuDiCa) technique (e.g., Guevara et al., 2024), enables this real-time discussion.

LuDiCa is a two-phase technique that draws inspiration from Whitehurst et al. (1988) dialogic reading cycle for children but adapts it to focus on deep comprehension and can be adapted for readers of all ages. The first phase of LuDiCa involves analyzing the narrative and formulating open-ended questions aimed at fostering comprehension, not just of the events in the story, but of their deeper connections, meanings and implications. This analysis encompasses Narrative Events and Narrative Functions. Narrative Events are the actions and happenings in the story. Narrative Functions, on the other hand, are especially important for deep comprehension, because they consist of thematic units that are logically interconnected giving meaning to the narrative. A Narrative Function does not coincide on a one-to-one basis with specific excerpts (Pfeiffer-Flores et al., 2020a) but is diffused throughout the narrative and can be expressed through story events, but also through descriptions, figurative language, stylistic strategies, voice, etc. In this sense, they are what the story expresses, what it shows. Some examples of Narrative Functions are story atmosphere, characters' feelings, thoughts and motivations, non-obvious connections between story events, metaphorical or symbolic expressions, and so on (to delve deeper into the concept of Narrative Functions, see Pfeiffer-Flores et al., 2020b). The open-ended questions are designed to foster discussions around important Narrative Functions.

The second phase of LuDiCa consists in applying the planned open-ended questions during shared reading, with scaffolding and encouragement oriented around the Narrative Functions. The facilitator's discourse remains under the joint control of these functions and the group's responses, gently steering discussion toward deeper comprehension without reducing it to either rigid answers or unfocused talk. Dialogic cycle of LuDiCa has shown promise in enhancing narrative comprehension across various audiences, including school-age children (e.g., Medeiros and Pfeiffer-Flores, 2017), autistic children (e.g., Caldas Souza and Pfeiffer Pfeiffer-Flores, 2020), autistic and neurotypical adolescents (e.g., Guevara et al., 2024), and adults (e.g., Moraes and Pfeiffer-Flores, 2022) and has also shown promise in helping elementary school teachers choose better questions and scaffold children's discussions more fruitfully, leading to better and deeper narrative comprehension (Bisello, 2018).

Guevara et al. (2024)'s results, although they did not directly focus on empathy, suggest that LuDiCa could provide a conducive context for empathy to emerge among participants. Their study assessed interactions between autistic and neurotypical adolescents during LuDiCa sessions, categorizing dialogues into conversational acts, self-talk, initiations, and questions. Results showed an increase in all speech categories during LuDiCa in comparison with Baseline (non-dialogic shared reading), for both neurotypical and neurodivergent participants, as well as in expressions of interest and mutual social reinforcement. Guevara et al. (2024) emphasized the mediator's role in creating an environment where participants feel safe to express their opinions and share personal experiences.

In light of the above, the purpose of this study was to investigate whether and how LuDiCa facilitates (1) expressions of empathy among participants (either as empathizer or empathizee); (2) expressions of literary empathy.

Contact with unfamiliar experiences allows for assessment of perspective taking because it requires an effort and an openness on the part of the reader toward the comprehension of events and Narrative Functions not immediately familiar to them. For this study, we chose the experiencers of refugee and immigrant children, an experience not immediately familiar to most participants, as told through a literary lens.

## Method

### Approval of the research project by the research ethics committee with human beings

The research project related to this study was approved by the Research Ethics Committee of the Institute of Human Sciences of the University of Brasília (CAAE: 67798123.1.0000.5540), and the participation of children was authorized by the school, by the guardians (informed consent), and by the children themselves (informed assent).

### Participants

Twelve children, aged 9 to 11 years, students of the 4th and 5th grades of elementary school in a public school in the Federal District, Brazil, participated in this experiment. Teachers were asked to nominate children for participation in the research based on the following inclusion criteria: regular attendance at school and demonstration of interest in participating.

At the school where the research took place, two children, one girl, and one boy, immigrants from Venezuela, were identified. Because they met the inclusion criteria and were within the appropriate age range for the study, they were invited to participate. The girl participated in Group 2 and the boy in Group 3 (the groups are explained below). For the formation of the groups, children from different school classes were mixed, prioritizing that child from the same class participated in different groups. In addition, Brazilian children from Groups 2 and 3 were asked if they knew the Venezuelan child participating in each group, to which they answered no. Thus, the participation of children from two different nationalities, including immigrant children, became an additional opportunity for the emergence of instances of empathy.

After the selection of participants, the children were divided into four groups, which went through the Baseline – BL and the experimental condition and were analyzed as subjects, as explained below.

The parents of the children were contacted and invited to attend an orientation meeting about the research. In this meeting, the objectives and procedures of the research were explained, as well as the voluntary nature of entry and participation in the research and the total freedom to choose whether or not to participate at any time. After that, signatures were collected on the Informed Consent Forms and on the Authorization Term for the Use of Image and Voice Sound for Research Purposes. Some parents were unable to attend, and with these, a phone call was made, and they were sent, via WhatsApp, an explanatory video, recorded by the researcher herself, with information about the research. For these cases, the terms were sent by the children, in their backpacks, so that the parents could sign them at home if they agreed to their children's participation.

The informed assent (Goldim et al., 2003) was obtained through an explanation, with language appropriate to the children's ages, about the purpose of the research and how the sessions would take place. It was explained that participation was voluntary and that they could stop participating at any time. In addition, a demonstrative session was held, using a children's literature work that would not be used in data collection. After the demonstration, the children's signatures were collected on the Informed Assent Form.

## Location

All sessions took place at the children's school. They were held in the room of the Specialized Learning Support Team. The room has a large rectangular table, which comfortably accommodated all the children and the mediator during the sessions and allowed the positioning of the video camera to capture all participants. During the sessions, only the children and the mediator were in the room; however, there were some interruptions, in specific sessions, by members of the school staff and/or because there was a lot of noise in the school environment. Immediately after the interruptions, the sessions were resumed, and whenever necessary, the part of the story and the dialogues raised were recalled.

## Materials/instruments

This data collection was made in the Portuguese language. Two works were used for data collection. For the BL sessions, the work “Valentes: Histórias de pessoas refugiadas no Brasil” (Brave: Stories of Refugees in Brazil), by Aryane Cararo and Duda Porto de Souza, with illustrations by Rafaela Vilela and cover art by Joana Amador, from Editora Schwarcz, was chosen. It is a reference work on the situation of refugees in Brazil and had the support of the United Nations Refugee Agency – UNHCR. The work brings information, statistical data, and accounts of experiences of refugees in Brazil, coming from Asia, Africa, Europe, and Latin America.

For this research, 12 refugee stories portrayed in the work were selected. The stories were used to form Interaction Cards. These were read to the children in the BL, in the Probe sessions, and in the Return to Baseline – RBL. The cards were excerpts from the true stories, self-reported by the refugees. Each Interaction Card contained between 3 and 4 paragraphs of the story of a refugee in Brazil, each one independent of each other.

For the intervention sessions, the work “O Cometa é um sol que não deu certo” (The Comet is a Sun That Didn't Succeed), authored by Tadeu Sarnento, with illustrations by Apo Fousek, was used. The work tells the story of children living in a refugee camp in a desert in Jordan, portraying the reality and common experiences in the location. The narrative is written in the third person, with an omnipresent narrator, and contains various streams of consciousness from the characters, emphasizing the psychological aspect of the story related to the events experienced by them.

The work was chosen for its literary nature and for containing experiences of children that are different from the experiences of most children participating in this research, allowing the emergence of instances of empathy. This was one of the few works that addresses the subject matter and is structured with chapters, enabling better observation of the reader's immersion in the text. Furthermore, it is an engaging work with quality narrative, attested by having won the Barco a Vapor Prize. It consists of 9 chapters, and each one was read, using the LuDiCa technique, in intervention sessions. All data collection sessions were recorded using a digital camera for subsequent analysis of interactions.

## Analysis of narratives and preparation of interventions for mediations

Both the narrative reports, taken from the work “Valentes: Histórias de pessoas refugiadas no Brasil”, and the chapters of the work “O Cometa é um sol que não deu certo” were previously analyzed in terms of Narrative Functions and Events (cf. Pfeiffer-Flores et al., 2020b). Each narrative read in the BL, probes, and RBL sessions contained between two and four Narrative Functions.

Regarding the work “O Cometa é um sol que não deu certo”, each chapter contains between four and seven Narrative Functions. For LuDiCa, the most important Narrative Function of each chapter was defined, and based on it, two questions were planned to serve as dialogical interventions in different parts of the chapter.

The analyses of four reports from the Interaction Cards (33.33% of the total cards) and three chapters (33.33% of the total chapters) of the work used in the intervention were subjected to agreement between judges, initially in a meeting of the Open Books Research Group^1^, for refinement and discussion of disagreements, and later, between the first author and two researchers with experience in Narrative Functions analyses. A percentage of 95.76% agreement was reached for the Interaction Cards and 94.44% for the work “O Cometa é um sol que não deu certo.”

## Procedure

### Design

A multiple baseline design across groups was used, with the use of probes. In a multiple-baseline design, the introduction of the intervention is intentionally staggered across participants, behaviors, or contexts to establish experimental control. Each baseline thus serves as a control for the others: if changes in the dependent variable occur only after the intervention is introduced, it becomes unlikely that external or coincidental factors account for the observed effects. The replication of this pattern across baselines strengthens the causal inference that the intervention, rather than extraneous variables, produced the observed change (Kazdin, 2011).

For this, the children were divided into three groups, each composed of four children. Since the focus of this research is on interaction, as the theoretical perspective of empathy explained in introduction, for data analysis, the group was considered as the subject, thus, behaviors were counted per group. Each group was analyzed as a subject and went through the following conditions:

Baseline: In the BL, Interaction Cards were used. The cards served as prompts for free interaction between the children and to verify their initial repertoire in relation to the empathy demonstrated by the person present in the narrative, as well as between them. Thus, it is a provocative material to check the children's initial empathic repertoire. The mediator read one card per session, in continuous reading (without interspersing it with questions for dialogues), and immediately after the reading, the mediator instructed the children to talk among themselves about the story read on the card while she filled out some forms in another table. Thus, the mediator did not participate in this interaction about the card read. When the children called, the mediator asked if they had all said everything they wanted to say, and if so, the session of the day was ended.

To avoid long BL, and consequently, prevent the unfamiliar theme from becoming familiar even before the intervention, probes were used. Probe sessions had the same format as the LB sessions, with the same interaction dynamics and use of Cards. Therefore, the beginning of the experiment, a BL session was held with all groups, but at this point, only Group 1 entered the actual BL, with continuous sessions.

While Group 1 continued in the BL sessions, the other groups did not participate in sessions. When Group 1 entered the intervention, Groups 2 and 3 were probed again (probe 2), and from then on, Group 2 entered the actual BL. When Group 2 entered the intervention, a new probe was conducted in Group 3, which then started the BL. The actual BL lasted for at least three sessions and had a difference of at least 2 sessions between one group and another. The achieved Narrative Functions were used as stability criteria. In this sense, BL Narrative Functions data were visually inspected for level, trend, and variability and, following Kazdin (2011), was considered stable when at least three consecutive data points showed no increasing or decreasing trend.

Experimental Condition: In the intervention, the work “O Cometa é um sol que não deu certo” was used, which was read dialogically, using the LuDiCa technique. For each session, two questions based on Narrative Functions were planned, with one Function addressed for each chapter. The mediator asked the planned question related to one of the Narrative Functions, and if the children had difficulty understanding and verbalizing the Function, the scaffolding procedure typical of LuDiCa (cf. Medeiros and Pfeiffer-Flores, 2017) was initiated by the mediator.

**Figure 1:**
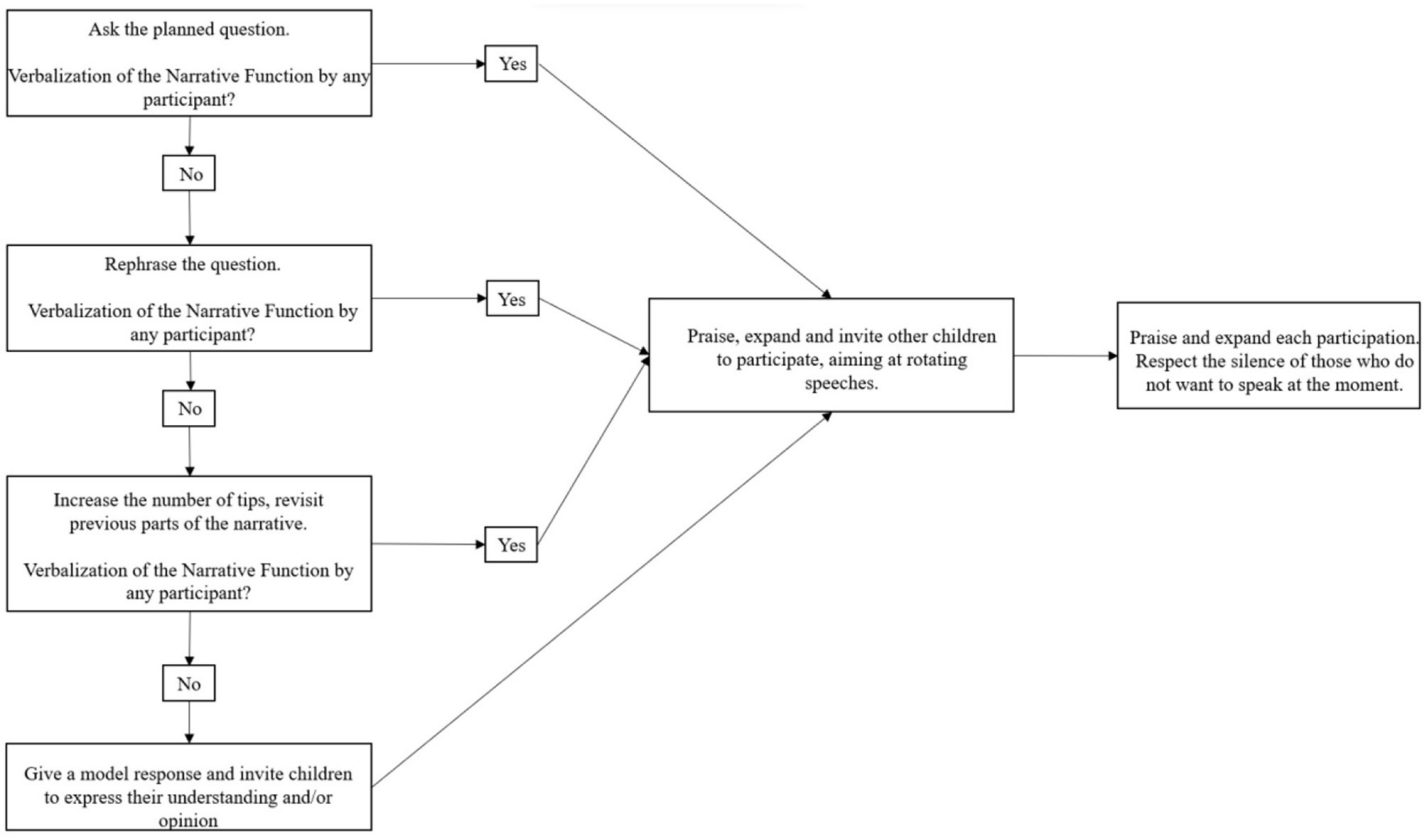
Diagram of the scaffolding performed by the mediator in the dialogical interactions with the children.

Thus, after the planned question, if the children did not verbalize the evoked Narrative Function, the mediator reformulated the question. If, after that, there was still no demonstration of understanding by the children, the mediator increased the number of hints, recalling previous narrative events. This process always occurred responsively, that is, based on the knowledge that the children already demonstrated and following their interest in the dialogue. Finally, if the Function was not verbalized, the mediator provided a response model and ensured the children's understanding. The mediator always rotated speech in the reading circles, that is, encouraged all children to participate in the dialogue. Figure 1 shows the scaffolding performed by the mediator in the LuDiCa sessions.

Thus, the independent variable was LuDiCa and the dependent variable was empathetic behavior, to be evaluated through behavioral measures, as explained below.

Probes and Return to Baseline: In addition to the initial probes conducted before the actual start of the BL, three probes per group were conducted throughout the intervention. This was because the contingencies present in the BL and intervention sessions were very different, and it was noticed that the BL contingencies could highlight other types of empathetic manifestation behaviors (for example, it was common in BL sessions for one of the children to take on the role of group mediator during free dialogue among them). Thus, the probes conducted during the LuDiCa sessions had the same format as the BL sessions. Similarly, two RBL sessions were conducted after the completion of reading the work. Table 1 shows the number of sessions each group underwent in each experimental condition.

**Table 1:** Number of sessions that each group underwent in each experimental condition.

**Group**	**Probe sessions**	**Baseline sessions**	**LuDiCa sessions**	**Return to baseline sessions**
Group 1	3	3	9	2
Group 2	4	4	9	2
Group 3	5	5	9	2

## Data collection

Data collection took place between April 9, 2023, and May 31, 2023. All sessions took place at the children's school three times a week. The timing for conducting the sessions was coordinated with the teachers to avoid interference with the classroom dynamics. Thus, on the designated days and times for the sessions, the mediator would visit the classrooms, gather the children from each group, and direct them to the room reserved for the interventions.

## Behavioral measures

Interactions were categorized into three categories and six subcategories of speech, as detailed below. The measures were derived from the functional analysis of participants' verbal behavior. All examples cited below to clarify behavioral measures are actual statements made during the sessions. To address the justification for the use of each measure, based on the concept of Literary and Interpersonal Empathy (see Rogoski, 2024).

### Category 1: literary empathy

Subcategory 1.1. Narrative Functions: Narrative Functions pertain to the psychological plane of the narrative, including dimensions such as the story's atmosphere, characters' feelings, characterizations, motivations, desires, and ideas conveyed throughout the narrative. These instances were previously listed based on the narrative's preliminary analysis. Subsequently, each session was analyzed in terms of the Narrative Functions spoken by the group, and they were quantified through an adjusted score of Functions achieved, as explained in the data analysis section.

Subcategory 1.2. Distancing: Distancing refers to any relationship between the story and the participants' own experiences, whether directly lived (e.g., personal experiences related to the story, such as “I learned to swim there in Ceará, in my grandfather's or grandmother's dam”) or indirectly (e.g., world knowledge related to the story, such as in the statement “Just like the people in Ukraine”; or family stories, such as “my grandma, she didn't date first and then get married, she got married straight away”).

### Category 2: interpersonal empathy

Subcategory 2.1. Completing Previous Speech: completion occurs when one child follows up or completes another child's speech (only between children). It was counted regardless of whether there was a direct request or not. That is, if one child is saying something and directly asks for help from another child (through a question directed at them, for example), this measure is counted. Additionally, if there is no direct request from the child, and still another child completes their speech, it is also counted as Completion. This includes responding to questions from other peers and expansions of previous speech (e.g., one child says “a better life” and another expands with “a much better life”). In this measure, the second speech (which is the completion) makes no sense without the previous speech.

Subcategory 2.2. Prompt and Social Reinforcement (Prompt + SR): This measure refers to encouraging other peers to speak, either in the form of a prompt (e.g., one child asking others “What did you like most about the story?”; or, for example, when one child shows hesitation in speaking and another child says “Just speak”), or in the form of social reinforcement, with quick agreements (e.g., “uh-huh,” “yeah”). This measure, as explained earlier, aims to encourage a peer to express themselves and speak more; thus, they are not statements that bring their own content. It is counted only when it occurs between children. Questions asked by one child to others are also counted as prompts.

### Category 3: engagement

Subcategory 3.1. Enthusiasm: speeches demonstrating excitement about the story (e.g., different speech topographies, such as acting out a play, imitating characters, or expressing excitement in speech) or interest in the story and the characters' reality (e.g., “I liked this story”; “This story is interesting”).

Subcategory 3.2. Initiations: These are speeches that initiate a dialogue or introduce a new topic. During readings, they are speeches (questions or comments) that interrupt the reading (e.g., a participant interrupts the reading and comments “oh, this has happened to me”). During a dialogue, initiations appear in the form of questions about the story (e.g., “Did this story really happen?”). They can be directed to both peers and the mediator.

Since they capture different aspects of interaction, subcategories from different categories (Literary Empathy, Interpersonal Empathy, and Engagement) may coincide in the same speech. For example, from the perspective of understanding the narrative, a child may be recounting a Function, and from the engagement perspective, this same speech may be an Initiation. Similarly, for the Engagement category, there may be this overlap between subcategories (e.g., a speech may be an Initiation demonstrating Enthusiasm).

## Data analysis

Data analysis was performed based on the quantification of occurrences of behavioral measures (excluding Narrative Functions) during the dialogic portions of each session. For this purpose, a Partial Interval Recording procedure was applied (cf. Sella and Ribeiro, 2018). The video recordings were reviewed, and the time corresponding to dialogic interaction, defined as the period following the reading of the text in which participants engaged in verbal exchanges about the narrative or related themes, was divided into consecutive 15-s intervals. Each interval was coded for the presence or absence of the target behaviors. The counting of intervals began immediately after the mediator stopped the reading; and finished immediately at the resumption of reading, marking the transition from reading to discussion and back.

After recording the occurrences in the spreadsheet, the percentage of intervals in which each behavior occurred was calculated per session. That is, the total number of intervals divided the number of intervals in which a behavior was observed in that session, and the result was multiplied by 100.

The Narrative Functions were quantified using an adjusted score based on the narrative's complexity. It was chosen to demonstrate the Narrative Functions based on narrative complexity because the Interaction Cards used in the BL, probes, and RBL were simpler than the chapters of the work “O cometa é um sol que não deu certo,” used in the intervention.

For the calculation of narrative complexity, the sum of Narrative Functions + Narrative Events was used (Text Complexity = Number of Functions + Number of Events). The normalized score of Narrative Functions achieved was calculated by multiplying the percentage of Functions achieved in each chapter by the chapter's complexity (Normalized Score = Percentage of Recognized Functions × Text Complexity). To standardize the measures presented in the graphs in terms of percentage, the normalized score was then adjusted based on the maximum possible score for the sessions. Thus, the adjusted score presented in the graphs refers to the normalized score divided by the maximum possible score of the session, with the result multiplied by 100 (Adjusted Score = [Normalized Score/Maximum Possible Score] × 100).

The effect size of LuDiCa was calculated using the non-parametric Tau-U AxB statistical test (Brossart et al., 2018) for all behavioral measures, with comparisons between the BL and the intervention, as well as between the BL and the RBL. For the calculation of Tau-U, the probe sessions were disregarded.

## Observer agreement

Eighteen videos (30% of the total), comprising six BL, two probe, and ten intervention sessions, were randomly selected for observer agreement assessment. The sample was analyzed by the first author and an independent observer. Initially, a joint analysis was conducted for the purpose of training the observer and refining the behavioral measures. Afterward, the videos were analyzed independently to calculate the agreement. The agreement index [calculated by the formula (agreements/agreements + disagreements) x 100] was 100% for the Narrative Functions achieved, 89.47% for Distancing, 84.84% for Completion, 83.33% for Prompt+SR, 100% for Enthusiasm, and 100% for Initiations.

## Intervention fidelity

Twelve videos (20%), comprising three BL, two probe, and seven intervention sessions, were independently analyzed by seven researchers from the Open Books Research Group to verify intervention fidelity. For all sessions in all conditions, it was considered that the mediator read with animation, good expression and created an affectionate and welcoming environment for the participants. For all BL and probe videos, the mediator did not interfere in the free dialogue among the children in any of the analyzed videos. Regarding the intervention, in 100% of occasions, she asked the planned questions for the intervention, reformulated the question when necessary, and expanded on the children's responses. Only in one analyzed video, it was considered that the mediator missed two opportunities to be responsive to the children.

## Results

### Literary empathy

**Figure 2:**
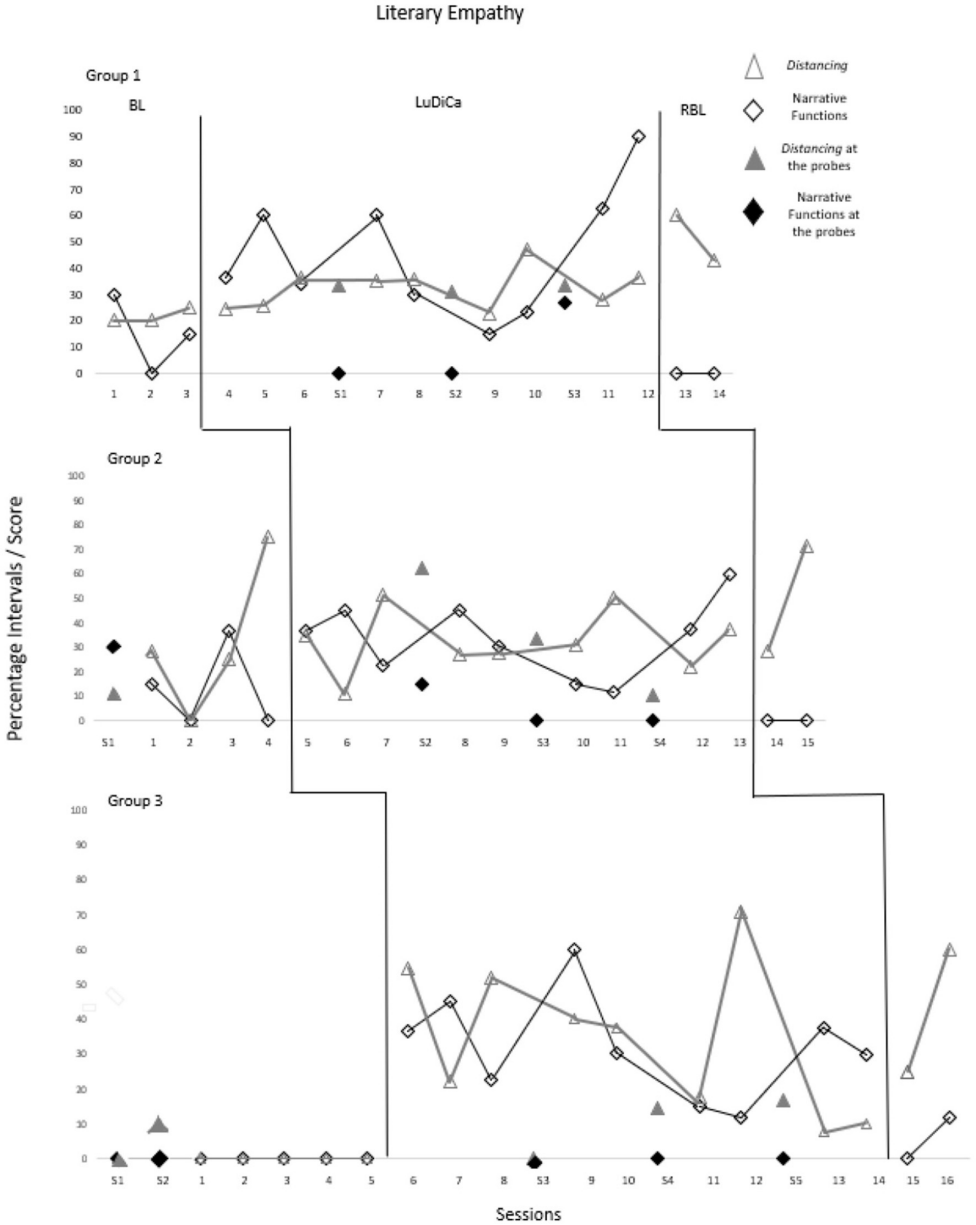
Percentage of intervals with distancing occurrences and narrative functions adjusted score. The sessions indicated by S1 to S5 refer to probes 1 to 5.

Figure 2 presents the percentage of intervals in which Distancing occurred and the adjusted score of Narrative Functions achieved throughout the sessions. Table 2 shows the Tau-U test results for these measures.

**Table 2:** LuDiCa effect size data on narrative functions and distancing by group.

**Tau-U**	**Conditions**	**Group 1**	**Group 2**	**Group 3**
Tau-U effect for narrative functions	BL × LuDiCa	0.77	0.91	1
	BL × RBL	−0.66	−0.33	0
Tau-U effect for distancing	BL × LuDiCa	0.77	−0.66	1
	BL × RBL	0.66	−0.16	1

BL, baseline; RBL, return to baseline.

Scores up to 0.65 = small effect; between 0.66 and 0.92 = medium effect; between 0.93 and 1.00 = large effect.

Children in Group 1 demonstrated greater Literary Empathy from the intervention. Despite this, expressions of Functions varied, especially in sessions 8 and 9, referring to chapters 5 and 6, in which they had more difficulty. From session 10, Literary Empathy improved again, with children achieving almost all Functions of the last chapter of the work (90% in adjusted score). Group 1 did not report Narrative Functions in the first two probe sessions, but they improved performance in the last one. Similarly, in the two RBL sessions, they did not express statements indicating narrative understanding.

Group 2 showed variation in the Narrative Functions achieved throughout all sessions. Still, performance in the intervention was better than in the BL, as there were no sessions without Functions achieved during LuDiCa. In the readings of chapters 5 and 6 (sessions 9 and 10), Literary Empathy suffered a setback. After that, the children began to demonstrate gains in the Functions achieved. In probe sessions interspersed with the intervention and in the RBL, few Narrative Functions were achieved (between 0 and 15%).

Children in Group 3 did not express Narrative Functions in the initial probes and in the BL. They began to demonstrate Literary Empathy from LuDiCa. Similarly to the other groups, here the children also had more difficulty in chapters 5 and 6 (sessions 10 and 11), after which they began to express more Narrative Functions again. In probe sessions interspersed with the intervention and in the first RBL session, they did not express Literary Empathy through Functions.

When excluding chapters 5 and 6 from the graphical analyses of Functions achieved, it is observed that all groups showed an increasing trend in this measure throughout the intervention. That is, the children demonstrated being more and more “inside” the work, expressing greater understanding and, consequently, a higher level of Literary Empathy. Additionally, all Narrative Functions of the work “O Cometa é um sol que não deu certo,” addressed through planned questions, were achieved in all sessions and by all groups.

Thus, the results of the three groups showed that LuDiCa has great potential to foster Literary Empathy, which is also evidenced by the effect size (Tau-U), large for Group 3 and medium for Groups 1 and 2.

Regarding Distancing, Group 1 consistently demonstrated more Literary Empathy from LuDiCa. They reported more personal experiences related to the story in probe sessions and in RBL, when compared to the BL, indicating a repertoire gain, presenting autonomy in interaction and placing themselves side by side with the characters without the need for an adult mediator. The Tau-U showed a medium effect of LuDiCa on the expressions of Distancing for this group.

Children in Group 2 expressed Distancing in a growing trend, already in the BL. With the start of LuDiCa, there were fewer speeches in which they placed themselves side by side with the characters, compared to the last BL session, but the percentages remained relatively constant throughout the chapters' readings in the intervention. By this pattern, the Tau-U result was negative. Children in this group reported fewer Distancing, throughout probe sessions interspersed with the intervention, which may indicate that they became accustomed to the presence of an adult mediator in interactions. On the other hand, upon entering the RBL, they reached 71.42% of Distancing, indicating potential to compare themselves to the characters even without the help of the mediator.

Children in Group 3 presented little repertoire of Literary Empathy in the BL, both for Narrative Functions (as explained above) and for Distancing. In LuDiCa, they reported personal experiences in all sessions, as well as in the last two probe sessions and in RBL. This, again, indicates a repertoire gain and autonomy in relation to the mediator, data corroborated by the Tau-U result, with a large effect of LuDiCa for this group.

### Interpersonal empathy

**Figure 3:**
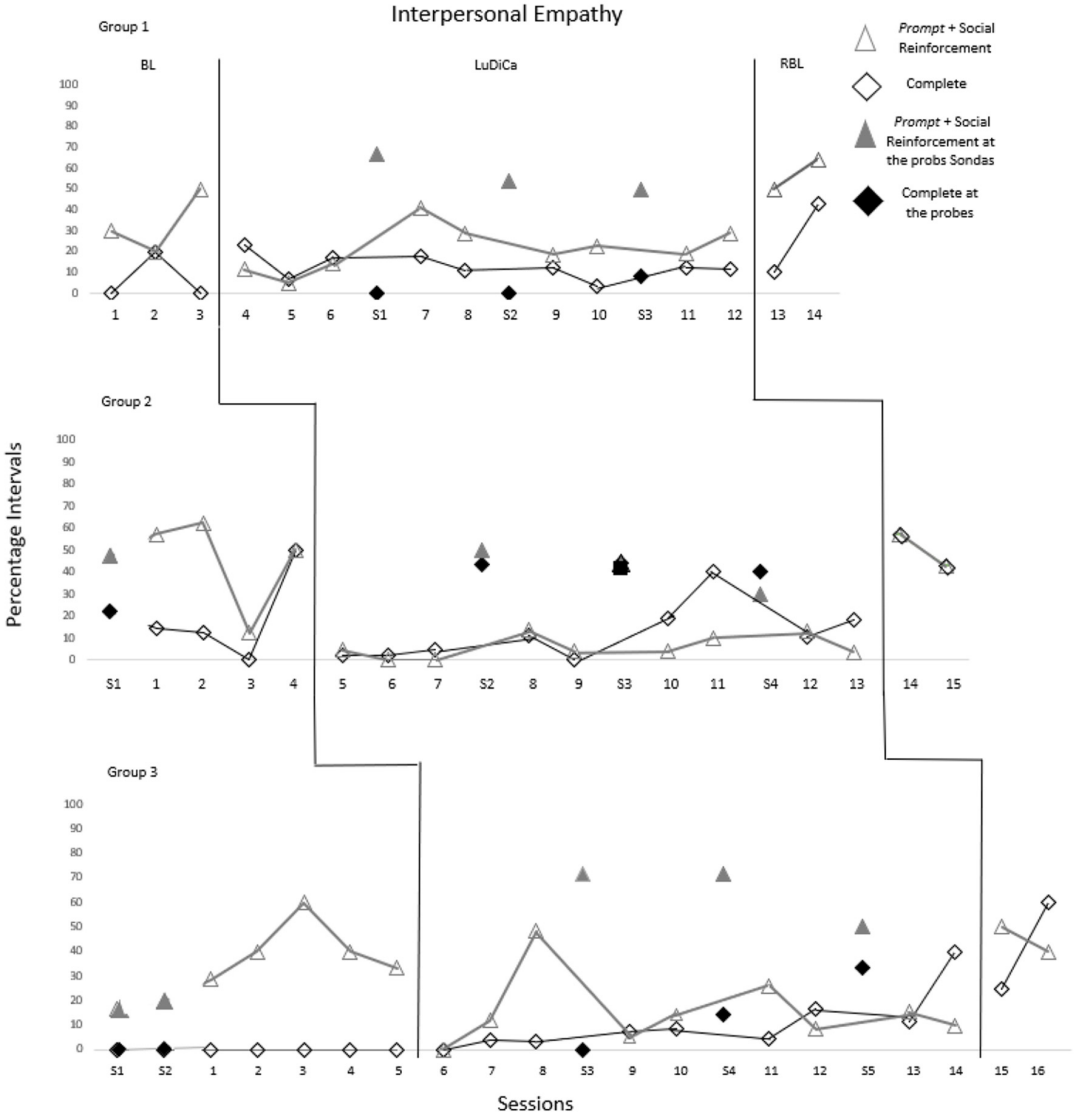
Percentage of intervals with prompt + SR and complete occurrences. The sessions indicated by S1 to S5 refer to probes 1 to 5.

Figure 3 shows the percentage of intervals in which Prompt+SR and Completing occurred throughout the sessions. Table 3 presents the results of the Tau-U test for these measures.

**Table 3:** LuDiCa effect size data on Prompt + SR and complete by group.

**Tau-U**	**Conditions**	**Group 1**	**Group 2**	**Group 3**
Tau-U effect for prompt + SR	BL × LuDiCa	−0.62	−1	−0.85
	BL × RBL	0.83	−0.12	0
Tau-U effect for complete	BL × LuDiCa	0.40	−0.19	0.66
	BL × RBL	0.66	0.75	1

BL, baseline; RBL, return to baseline.

Scores up to 0.65 = small effect; between 0.66 and 0.92 = medium effect; between 0.93 and 1.00 = large effect.

Regarding Prompt + SR, children from all three groups already demonstrated this repertoire in the BL. In Group 1, performance remained stable during LuDiCa. However, in Groups 2 and 3, BL performance was better than in LuDiCa. The negative results of Tau-U demonstrate this trend.

On the other hand, in the sessions of probe interspersed with the intervention and in RBL, all groups used more Prompt + SR than in the intervention, with Group 3 also showing this gain compared to BL. This indicates that this is a repertoire better utilized in the absence of the mediator, moments in which children take on this role among themselves.

Completing had a similar pattern to Prompt + SR. Groups 1 and 2 already demonstrated this repertoire in the BL, albeit in low percentages, which remained stable during LuDiCa. This was evidenced by a small effect of Tau-U for these groups. Children in Group 3, on the other hand, did not complete each other's sentences in the initial probes or in the BL. They started doing so from LuDiCa, as evidenced by a medium effect of Tau-U.

For all three groups, especially Groups 2 and 3, Completing was more expressed in probes interspersed with the intervention and in RBL. This indicates that this is also a repertoire favored by the absence of the mediator, as during these moments, children took the lead in completing each other's sentences, helping or confirming their peers' speech.

### Engagement

**Figure 4:**
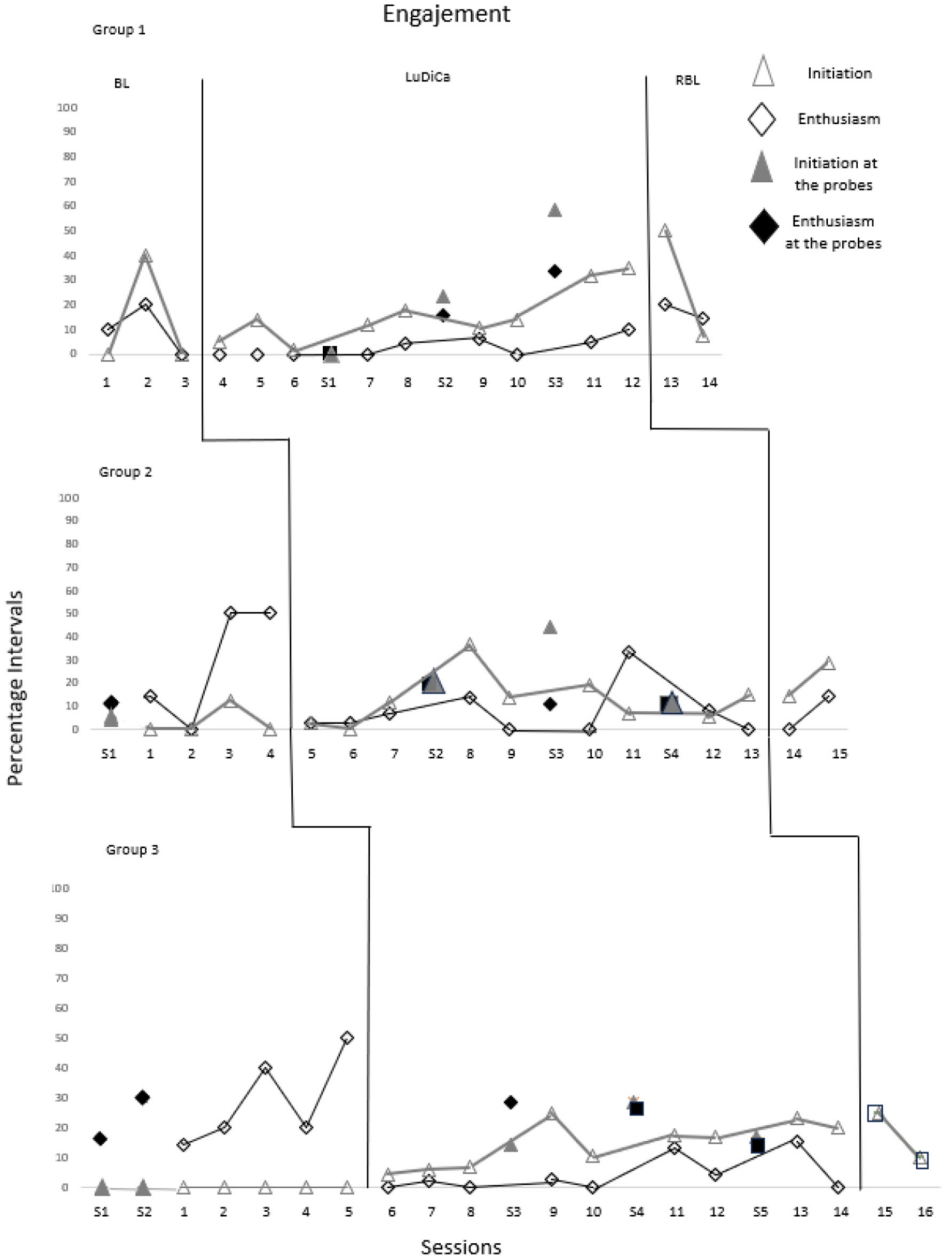
Percentage of intervals with occurrences of initiations and enthusiasm. The sessions indicated by S1 to S5 refer to probes 1 to 5.

Figure 4 presents the percentage of intervals in which Initiations and Enthusiasm occurred throughout the sessions. Table 4 shows the results of the Tau-U test for these measures.

**Table 4:** LuDiCa effect size data on Initiations and Enthusiasm by group.

**Tau-U**	**Conditions**	**Group 1**	**Group 2**	**Group 3**
Tau-U effect for initiations	BL × LuDiCa	0.33	0.63	1
	BL × RBL	0.66	1	1
Tau-U effect for enthusiasm	BL × LuDiCa	−0.51	−0.51	−1
	BL × RBL	0.50	−0.83	−1

BL, baseline; RBL, return to baseline.

Scores up to 0.65 = small effect; between 0.66 and 0.92 = medium effect; between 0.93 and 1.00 = large effect.

Group 1 demonstrated more Engagement through Initiations in the LuDiCa, with an increase throughout the chapters and even in the RBL. In the last two probe sessions, they initiated more dialogues than in the intervention. Group 2, despite the variation, also initiated more dialogues in the LuDiCa than in the BL, a pattern maintained in the probes interspersed with the intervention and in the RBL. Group 3, on the other hand, did not initiate dialogues in the initial probes or in the BL. In the LuDiCa, they showed a growing trend of Initiations throughout the chapters, which was maintained in the probes interspersed with the intervention and in the RBL. These results are evidenced by the Tau-U calculations: small effect for Group 1, medium for Group 2, and large for Group 3.

Regarding Enthusiasm, the three groups showed less enthusiasm in the LuDiCa compared to the BL and probes. The way this behavior was operationalized favored its expression in interactions without the presence of the reading mediator, as evidenced by the negative Tau-U results.

## Discussion

The present study aimed to investigate the potential of LuDiCa in promoting demonstrations of Interpersonal and Literary Empathy. The context of reading circles, when interactive, such as LuDiCa, has been pointed out in previous studies (e.g., Fernandez-Quintanilla, 2020; Kurcikova, 2019) as conducive to this verification by allowing contact with the narrative and the experience of characters, which may be distant from that of the reader, as in the present study; as well as contact with the other, in the dialogue around the book.

Among interactive readings, LuDiCa appears to be even more conducive to the emergence of instances of Interpersonal and Literary Empathy, as it starts from a careful analysis of the work, composed of characters, their actions, emotions, feelings, motivations, etc., based on Functional Units, Narrative Functions, and Narrative Events (Pfeiffer-Flores et al., 2020b). Narrative Functions allow for the planning of interventions that foster Literary Empathy.

In this sense, this study demonstrated that LuDiCa is a technique that can foster the emergence of instances of Interpersonal and Literary Empathy. Also, the single-subject experimental design allowed verification of the process of interactions over the sessions. The increase in Narrative Functions, Distancing, and Completing throughout the sessions, demonstrating the effect of the intervention, showed that Interpersonal and Literary Empathy can go hand in hand, although they do not always occur simultaneously. Thus, contact with characters from stories has the potential to foster empathy for the other. In the reading circles of the present research, sensitivity to the characters gave way also to sensitivity to the peers.

The data analysis based on the functional analysis of children's verbal behavior with subsequent quantification of behavioral instances allowed a more detailed view of the reader's experience in interaction with the text and of the participants in the reading circle in interaction with each other [cf. recommendations by Fernandez-Quintanilla (2020)]. This is one of the contributions of the present study, since previous studies resorted to static measures, such as questionnaires (e.g., Igartua et al., 2018; Vezzali et al., 2014), which do not demonstrate the complexity of interaction and emotional reactions to the text and the other in a reading circle.

Therefore, three groups, each composed of four children, went through LuDiCa sessions, using a narrative that brought experiences of children who lived in a refugee camp, experiences that are very different from those of most participating. The present study also provided the interaction of Brazilian children with immigrant children from Venezuela who had recently arrived in Brazil.

The data indicated that, in general, all three groups showed gains in terms of Narrative Functions, Distancing, Completions, and Initiations in their performance in the intervention compared to the BL. In other words, the children showed better performance in Literary Empathy, as well as instances of Interpersonal Empathy and Engagement, from LuDiCa. On the other hand, measures of Prompt + SR and Enthusiasm did not show significant differences in the intervention compared to the BL.

One potential limitation of the study concerns the selection of participants. The children were nominated by their teachers, based on criteria such as regular school attendance and willingness to participate. This might suggest a possible bias in the sample; however, the use of multiple baselines by groups eliminates this bias. This is because, in this type of design, the subject is compared to themselves. To achieve this, various behavioral measurements are taken of the same subject (in this case, the group) throughout the data collection period. In the data from this study, there was no ceiling effect (which, incidentally, would have made the intervention unfeasible). In this sense, even if there might have been a predisposition to expressions of empathy in this sample of participants, they still demonstrated growth in these behaviors.

Another issue that could be raised is related to the group level interaction analyzed. In the present study, the group was adopted as the unit of analysis because empathy was conceptualized not as an internal disposition, but as an occurring in interaction [as detailed explained by Pfeiffer-Flores and Rogoski (2023)]. Within this framework, empathic understanding and expressions are co-constructed as participants engage with one another and with the narrative. The behaviors coded as empathic were therefore understood as collective phenomena rather than as a sum of individual acts.

We acknowledge that behavioral measures could, in principle, have been conducted at the individual level. However, doing so would have implied a conceptual shift toward viewing empathy as an internal attribute manifested externally by individuals, which contrasts with the theoretical perspective taken here. This, of course, does not preclude future research from incorporating individual behavioral measures in addition to group measures. Future studies could also incorporate measures of nonverbal behaviors that express empathy (e.g., gestures, eye contact), as they are an important way of showing empathy.

Below are more specific analyses of each of the categories of behavioral measures.

### Literary empathy

The improvement in narrative comprehension, from LuDiCa, is a consistent finding in previous research by the Open Books Research Group, including different audiences, such as school-aged children (e.g., Medeiros and Pfeiffer-Flores, 2017), adults in Youth and Adult Education (Moraes and Pfeiffer-Flores, 2022), autistic children (Caldas Souza and Pfeiffer Pfeiffer-Flores, 2020).

It was observed that, regarding the achieved Narrative Functions, all groups had more difficulty in understanding chapters 5 and 6 of the book. For the purpose of understanding this occurrence, an analysis of the literary work was carried out, in terms of passages using literary or poetic language (use of metaphors, figures of speech, etc.) vs. literal language in all chapters. All of them present a greater amount of literary language when compared to literal language. However, chapters 5 and 6 are the ones that have a higher proportion of literary language compared to literal language.

These chapters present a greater flow of consciousness of the characters. In chapter 5, the main character demonstrates many concerns related to living in a refugee camp, the dangers he faces, his social relationships, and, mainly, how they would leave the camp after being expelled by the guards. This is demonstrated in the passage: “Of course he was also worried. He didn't know if he and his family would be able to cross the desert and, if they were successful, if the crossing by sea would work out and, if so, if they would be accepted in the new country on the other end, and they said it started on a very beautiful beach” (p. 61).

Chapter 6 is probably the most important for understanding the work because it is where the title is explained. This was initially difficult for the children to understand because it is metaphorical. However, when they understood the analogy of comets with refugees, the dialogues focused around this understanding, ceasing to report the other functions of this chapter. This analogy is well represented by the following passage: “- We are here in the refugee camp, right? And each one of us is a comet, that is, a sun that didn't succeed, as you yourself said (...). We are wandering, we are in transit, my good Emanuel. We will find our place, the place where we will succeed” (pp. 77–78). The focus of the dialogue in this Narrative Function indicates the children's engagement with the work and the reality of refugees.

In chapters 5 and 6, the tendency of decrease in the achieved Narrative Functions was accompanied by the results of Distancing. In other words, placing oneself alongside the character, expressing one's own experiences, was also hindered. However, in these occasions, Interpersonal Empathy remained stable or even increased, for example, in the emission of Completing behaviors by Group 2. This result goes against previous results, which measured interpersonal empathy in a static way, through questionnaires (e.g., Igartua et al., 2018; Vezzali et al., 2014).

These traditional studies start from a cognitivist view of empathy, in which the assumption is that by coming into contact with other worlds, through indirect contact (as in narratives), Interpersonal Empathy increases. The present result demonstrates that this does not always happen and/or does not happen immediately. The increase in Completing in Group 2 may indicate, moreover, that faced with difficulties in understanding the narrative, the children helped each other more, more frequently completing each other's sentences.

The increasing trend of results of Narrative Functions, from LuDiCa (when chapters 5 and 6 are disregarded), was an expected trend, as narrative comprehension increases with reader proficiency (Rogoski, 2024). The children in the present study had never had contact with long and complex narrative works, with rich details and experiences of characters so different from theirs. LuDiCa, in this sense, provided the development of greater reading proficiency, by emphasizing Narrative Functions, with techniques to foster deep comprehension. In addition, all groups achieved all Functions worked on in dialogic interventions, in all sessions.

Regarding Distancing, the increase in expressions, for all groups, from the intervention, indicates an improvement in the repertoire of Literary Empathy, as by going beyond the story's boundaries and bringing personal experiences, the children placed themselves side by side with the characters. This movement is analogous to the second level of the empathic phenomenon, described by Stein (1989), which occurs precisely when the empathizer stands side by side (without fusion of selves) with the empathized, after coming into contact, primarily, with their emotion, in the interaction.

Thus, in the first moment, the children came into contact, primarily, with the experience of the characters, from the interaction with the work and the understanding of the narrative. In the second moment, they deepen this contact, from imagination or reflection of “how if” they had already experienced that. In this way, they made the movement of placing themselves alongside the characters, bringing personal experiences in which they felt similarly, demonstrating understanding of those feelings and empathizing with them.

### Interpersonal empathy

The improvement in the Completing performance demonstrated an increase in Interpersonal Empathy. Completing the other's speech indicates that the children were attentive to them and, more importantly, sensitive to their needs. And this was facilitated by the reading circles, using LuDiCa. It was common for occurrences in which one child showed insecurity to say something and another child encouraged or helped them, completing their speech.

The contingencies presented in the BL, in the probes, and in the RBL were the same. By showing an increase in the probes intercalated with the intervention, compared to the BL, they demonstrated that possibly the mediator served as a model in the intervention sessions, by asking questions, validating answers, and giving positive feedback to the participants. In the intervention sessions, however, the mediator took the lead in this type of interaction, which decreased the opportunities for the children to present these behaviors directed toward each other, which was the way this measure was considered in the data analysis (occurrences only between children). In this sense, it may be interesting to think about strategies to decrease the centrality of the mediator's role, giving more opportunity for the children to have this function as well. Taking pauses and waiting for their input may be a way to start seeking solutions for this in future studies.

The increase in Prompt + SR, even only in the absence of the mediator, demonstrates an increase in Interpersonal Empathy. In other words, the children started to show more interest in what the others were saying, their point of view. By issuing more prompts to their peers, the children indicated that their opinions were important. And, by emitting social reinforcement behaviors, children validated and gave feedback to their peers' speeches, demonstrating interest in and sensitivity to the other.

Although there was no improvement in Prompt + SR performance when comparing BL data with the intervention, in Groups 2 and 3, the improvement in the probes intercalated with the intervention, when compared to the BL, indicates that the Empathy directed toward Venezuelan children may have increased. Furthermore, the subtle increase in Completing percentages in these groups follows the same direction of indicating an increase in sensitivity toward immigrant participants.

### Engagement

Initiations demonstrate a gradual increase in engagement in the activity and in the stories read, especially with the entry into LuDiCa. During reading, Initiations were demonstrated through interruptions, which was considered very positive, as it demonstrated attention to and curiosity about the story.

During dialogues, Initiations appeared as questions about the story. The narrative, presenting experiences far removed from the participants' own, encouraged them to show genuine interest and engagement. The children wanted to know more about these realities, asked questions, expressed concerns, or sought solutions to the characters‘ problems (cf. Gosen et al., 2015). Even the Venezuelan children had not had experiences very similar to those of the characters in the narrative. They understood the context of immigration, but demonstrated curiosity about living in a refugee camp in the middle of the desert (as was the case with the characters).

These are demonstrations of Empathy, as they are manifestations of interest in the life of the other, even if this is a character. It is very common, in the circumstances of current life, for people not to be interested in knowing and understanding the reality of the other, as this is an act of commitment, often not allowed by current circumstances (cf. Rifkin, 2009). In a world marked by the acceleration of time and the fluidity of social bonds, relationships tend to become increasingly transient and utilitarian, leaving little space for genuine engagement with the experiences of others (Bauman, 2004). In such a context, empathic understanding requires an intentional effort to resist indifference and to sustain the kind of dialogic openness that contemporary life so often discourages.

A similar pattern to that found in Prompt + SR was observed regarding the measure Enthusiasm. There were better performances of the three groups in the BL, probes, and RBL sessions. This is because this measure was probably more evoked by the contingencies of these sessions, compared to intervention sessions. In this case, it was observed that, faced with the instruction given by the mediator at the end of the reading (“now it's your turn to talk, among yourselves, about the story read”), it is common practice, in the verbal community, for the speaker to start this task by reporting their personal opinion about the story, especially if they liked it or not. Subsequent analysis of the videos indicated that the children commonly showed interest in the narratives, verbalizing that they liked the story, and consequently, demonstrating Enthusiasm.

In addition, when having a dialogue without the guidance of an adult mediator, the children tended to show more creativity, using different topographies of speech to communicate something about the story (e.g., using objects on the table to represent characters and enact a piece of the story; imitating the characters when retelling a piece of the story etc.). Although it appeared more in sessions with the BL format, this measure demonstrates that the children were attentive to the stories of immigrants and refugees, understood them, and, when given opportunities, showed interest in them. Consequently, they demonstrated Empathy.

The results of the present research thus demonstrated the possibility of engagement in deep and meaningful dialogues through contact with diverse experiences in narratives, indicating a way to foster less individualistic practices and more focused on the other. This implies that playful and educational strategies that encourage reflection and discussion of emotions aroused by reading may be important for cultivating Empathy not only regarding fictional characters but also regarding the reality of the other, in interpersonal interactions.

## Data Availability

The raw data supporting the conclusions of this article will be made available by the authors, without undue reservation.

## Footnotes

1 The Open Books Research Group is a research group at the University of Brasília. Linked to university extension activities, the group takes LuDiCa to public schools in the Federal District, Brazil.
